# The inhibition of cordycepin on cancer stemness in TGF-beta induced chemo-resistant ovarian cancer cell

**DOI:** 10.18632/oncotarget.22951

**Published:** 2017-12-05

**Authors:** Chia-Woei Wang, Bao-Hong Lee, Chen-Jei Tai

**Affiliations:** ^1^ Graduate Institute of Clinical Medicine, College of Medicine, Taipei Medical University, Taipei 11042, Taiwan; ^2^ Department of Obstetrics and Gynecology, School of Medicine, College of Medicine, Taipei Medical University, Taipei 11042, Taiwan; ^3^ Department of Traditional Chinese Medicine, Department of Internal Medicine, Taipei Medical University Hospital, Taipei 11042, Taiwan; ^4^ Traditional Herbal Medicine Research Center, Taipei Medical University Hospital, Taipei 11042, Taiwan

**Keywords:** cancer stem cells, chemotherapy, chemoresistance, cordycepin, ovarian cancer

## Abstract

Chemotherapy is one of the main approach for ovarian cancer. Cancer stem cells (CSCs) escape chemotherapy and lead to chemoresistance. We previously demonstrated that cordycepin (Cd) inhibited metastasis in human ovarian carcinoma cells, the aim of this study is to investigate the effects of Cd on ovarian cancer stemness. TGF-beta was used to induce chemoresistance of chemotherapeutic agent cisplatin in SKOV-3 ovarian cancer cells. After treating with 100 μM of Cd, cell viability, the percentage of cancer stem cells, and the levels of matrix metalloproteinases (MMPs) were decreased in TGF-beta-induced SKOV-3 cells. Treatment of Cd recovered E-cadherin levels and inhibited vimentin levels while TGF-beta treatment significantly increased the expression of vimentin and PGC-1alpha, and decreased E-cadherin levels in SKOV-3 cells, indicating that the action of Cd on cancer stemness may contribute to the regulation of epithelial-mesenchymal transition (EMT). Cd efficiently attenuated chemoresistance caused by TGF-beta in SKOV-3 cancer stem cells to promote the cytotoxicity of cisplatin.

## INTRODUCTION

Ovarian cancer is a common gynecological cancer and has the highest mortality rate worldwide. More than 70% of ovarian cancer cases have been diagnosed to be in the advanced stages; nonsurgical therapies like chemotherapy and radiotherapy are the main approaches for treating patients with ovarian cancer [1]. Ovarian cancer is a heterogeneous disease categorized into three subtypes: epithelial carcinomas, stromal carcinomas, and germ cell tumors [2]. Epithelial ovarian carcinomas account for approximately 85%–95% of all ovarian cancer cases [3, 4]. The invasive activity of epithelial tumor cells is based on single-cell migration such as mesenchymal-type movement [5].

Cancer stem cells (CSCs) represent a distinct subpopulation of the tumor cells that play important roles in the tumor initiation, progression, metastasis, chemoresistance, and relapse [6]. A recent *in vitro* experiment indicates chemoresistant effect was stronger in cisplatin- or paclitaxel-treated epithelial ovarian CSCs than in their differentiated progeny [7]. It is believed that the cytotoxic effects of chemotherapy are affected on most cells in tumor tissue but CSCs are still leave behind.

It has been found that CD44- and CD117-expressing epithelial ovarian cells isolated from primary human ovarian tumors are highly tumorigenic and capable of reestablishing their original tumor hierarchy when injecting into the nude mice that have been propagated with the original tumors [8], suggesting that the CD44^+^/CD117^+^ cells possess the properties of CSCs. Moreover, it has been found that CD133 and CXCR4 were expressed in ovarian CSCs isolated from ovarian OVCAR-3, -4, and -5 cells [9]. CD133 is the human homologue of mouse Prominin-1, a five transmembrane glycoprotein domain and a cell surface protein originally found on neuroepithelial stem cells in mice [10].

Cordycepin (3′-deoxyadenosine) display antitumor activity that has been shown antiangiogenic, antimetastatic, and antiproliferative effects, as well as inducing cancer cell apoptosis [11–14]. Moreover, the inhibitory effects of cordycepin on metastaisis and tumor size have been evaluated *in vivo* [15]. Recently, we demonstrate that cordycepin inhibits metastasis through down-regulatiing mitochondrial activity of estrogen-related receptor in human ovarian carcinoma cells [16]. Due to CSCs has associated with chemoresistance in cancer therapy and the effects of cordycepin on CSCs remain unknown, hence we evaluated the interference of cordycepin with or without chemotherapeutic durg cisplatin on CSCs formation caused by transforming growth factor-beta (TGF-beta) treatment in SKOV-3 human ovarian carcinoma cells.

## RESULTS

### The effects of TGF-beta on chemoresistance in ovarian cancer cells

**Figure 1:**
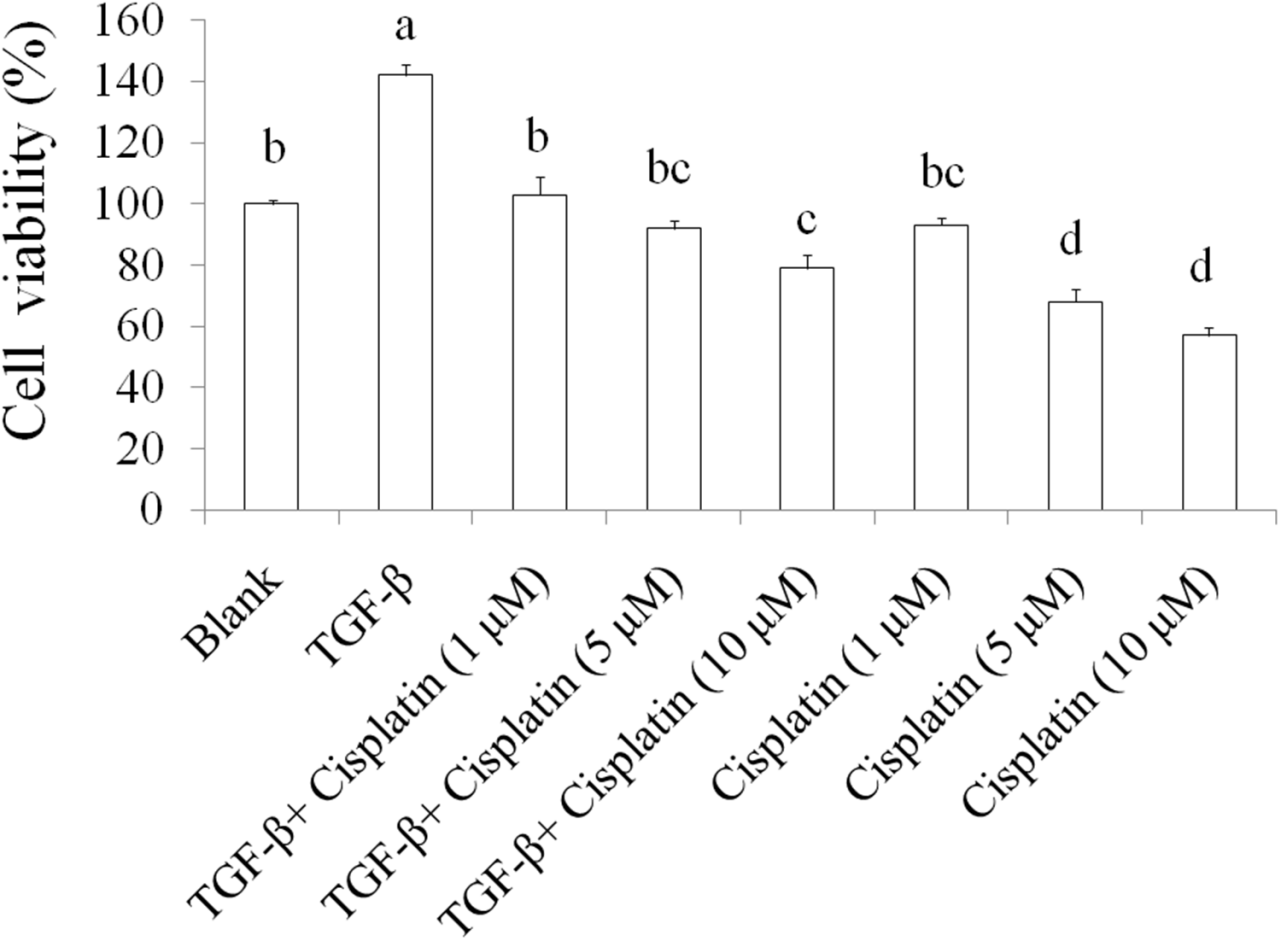
The effects of TGF-beta on chemoresistance in SKOV-3 cancer cells The SKOV-3 cells were treated by TGF-beta (20 ng/mL) for 5 days to cancer stemness induction, then these cells were sorted for CD44^+^CD117^+^ by flowcytometeric. Subsequently, SKOV-3 with CD44^+^CD117^+^ cancer cells were treated with cisplatin for 24 h thereby measuring survival cells by crystal violet. Data was shown by mean ± SD (n = 3). **(a.b.c.d)** values with one different letter superscript are significantly different from each other (p<0.05).

TGF-beta plays important roles on cancer stemness and chemoresistance [17, 18]. Inhibiting TGF-beta signals enhance the chemotherapy efficacy of breast cancer [19]. In this study, we used TGF-beta (20 ng/mL) to induce chemoresistance in SKOV-3 ovarian cancer cells and the results were shown as Figure 1, 5-day TGF-beta treatment significantly elevated cell viability while chemotherapeutic drug cisplatin (1, 5, and 10 μM) inhibited the cell viability in SKOV-3 cancer cells when comparing to the blank group. However, the suppression of cisplatin on cell viability was abolished by TGF-beta treatment in SKOV-3 cancer cells. These data indicated that TGF-beta induction markedly resulting in chemoresistance in SKOV-3 cancer cells.

### The effects of cordycepin (Cd) on cancer stemness induced by TGF-beta in ovarian cancer cells

**Figure 2:**
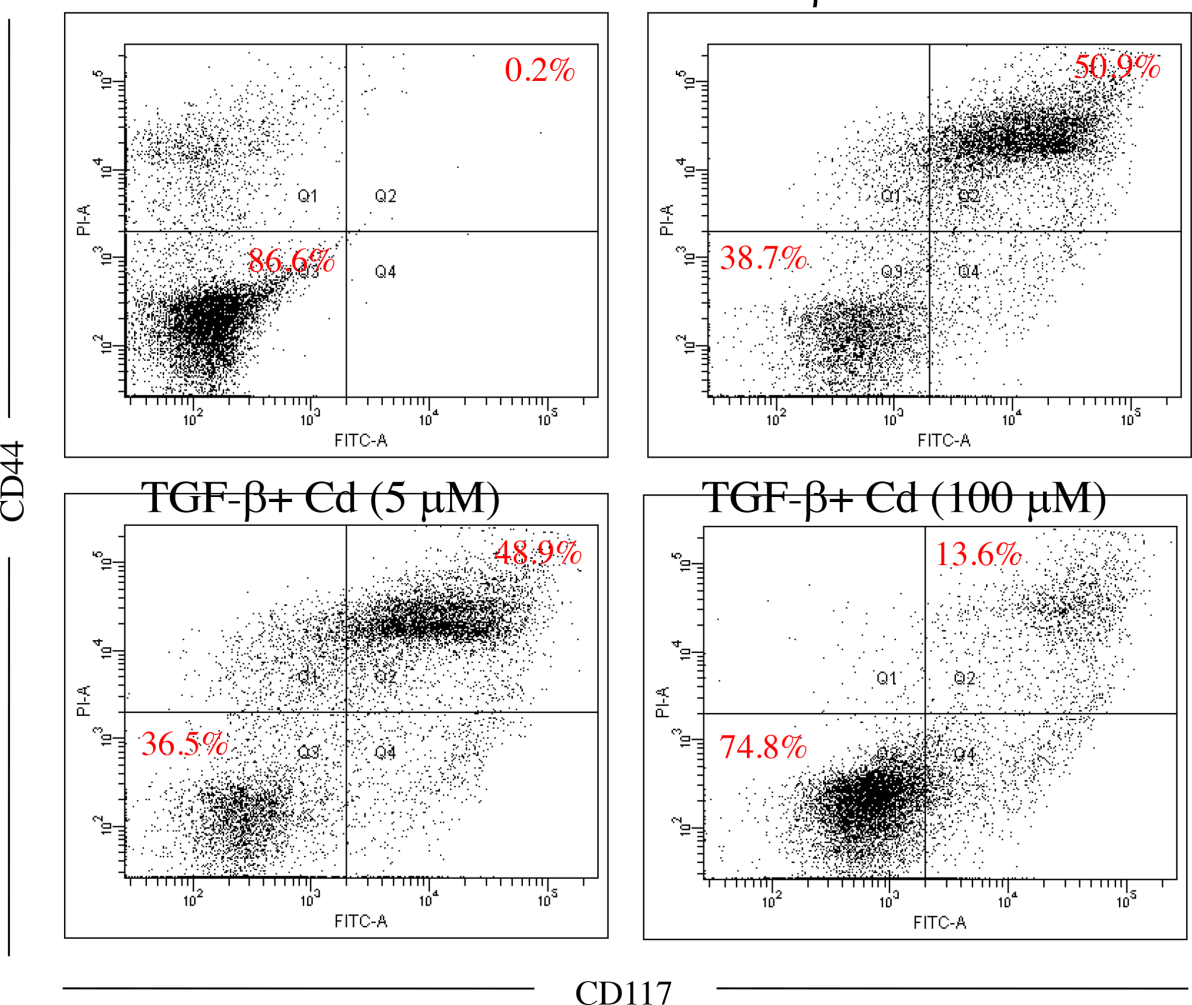
The effects of cordycepin (Cd) on cancer stemness induced by TGF-beta in SKOV-3 cancer cells Cells were treated with TGF-beta (20 ng/mL) every 24 h for five times (5 days) with or without Cd. And Cd treatment decreased the percentage of cancer stem cells (CD44^+^/CD117^+^) in ovarian SKOV-3 cancer cells. Data was repeated for 3 times.

**Figure 3:**
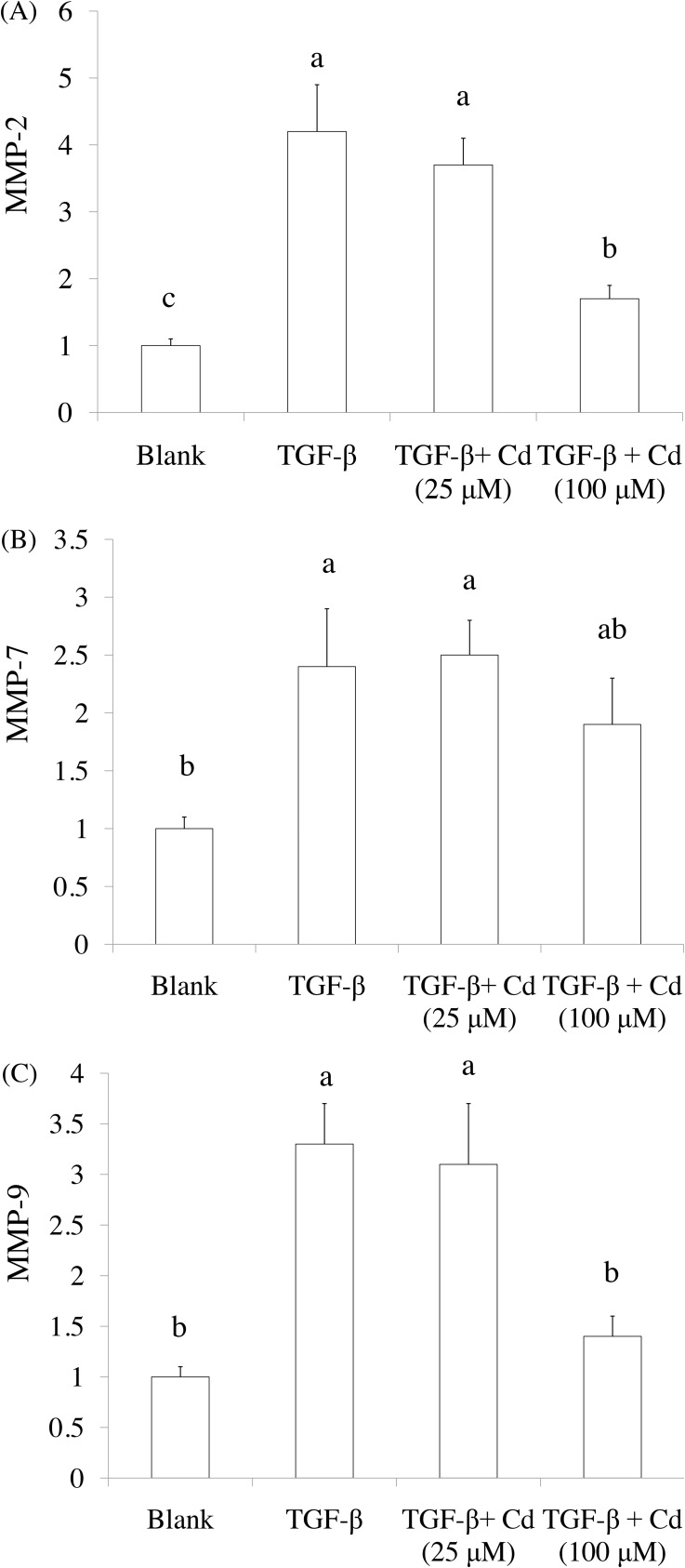
The effects of cordycepin (Cd) on MMP-2, MMP-9, and MMP-7 expression in TGF-beta-induced cancer stem cells by real-time PCR SKOV-3 cells were treated with TGF-beta (20 ng/mL) every 24 h for five times (5 days) with or without cordycepin (Cd). Data was shown by mean ± SD (n = 3). **(a.b.c)** values with one different letter superscript are significantly different from each other (p<0.05).

Both CD44 and CD117 were used as tumorigenic and stemness indicators in ovarian cancer cells [8, 20], SKOV-3 cancer stem cells were confirmed by CD44^+^ (positive) and CD117^+^ (positive) selection. As shown in Figure 2, 5-day induction of TGF-beta significantly elevated CD44^+^ (positive) and CD117^+^ (positive) population in SKOV-3 cancer cells. In addition, 100 μM of Cd treatment potentially reduced stemness of SKOV-3 cancer cells caused by TGF-beta. Matrix metalloproteinases (MMPs) including MMP-2, MMP-7, and MMP-9 are known to promote tumor progression and cancer stemness [21]. We found that TGF-beta treatment significantly increased the levels of MMP-2, MMP-7, and MMP-9 while Cd treatment decreased MMPs levels (Figure 3).

**Figure 4:**
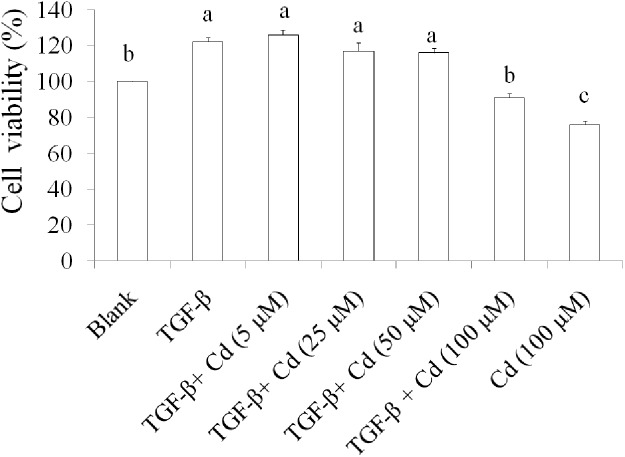
The effects of cordycepin (Cd) on cell proliferation in TGF-beta-induced cancer stem cells SKOV-3 stem cells (CD44^+^/CD117^+^) were collected and treated with TGF-beta (20 ng/mL) every 24 h for five times (5 days) with or without Cd. Data was shown by mean ± SD (n = 3). **(a.b.c)** values with one different letter superscript are significantly different from each other (p<0.05).

We recently reported that 200 μM of Cd induces cell death in SKOV-3 cells [16]. Therefore, low toxic dosage of Cd (100 μM) was chosen to investigate the effect of Cd on stemness in TGF-beta-induced SKOV-3 cancer cells. Results suggested that the cell viability in TGF-beta + Cd treatment group was higher than that in Cd treatment group, but lower than TGF-beta treatment group (Figure 4). These findings demonstrated that Cd has potential for suppressing stemness of TGF-beta-induced SKOV-3 cancer cells.

### Regulation of epithelial-mesenchymal transition (EMT) by Cd in TGF-beta-induced SKOV-3 cancer cells

**Figure 5:**
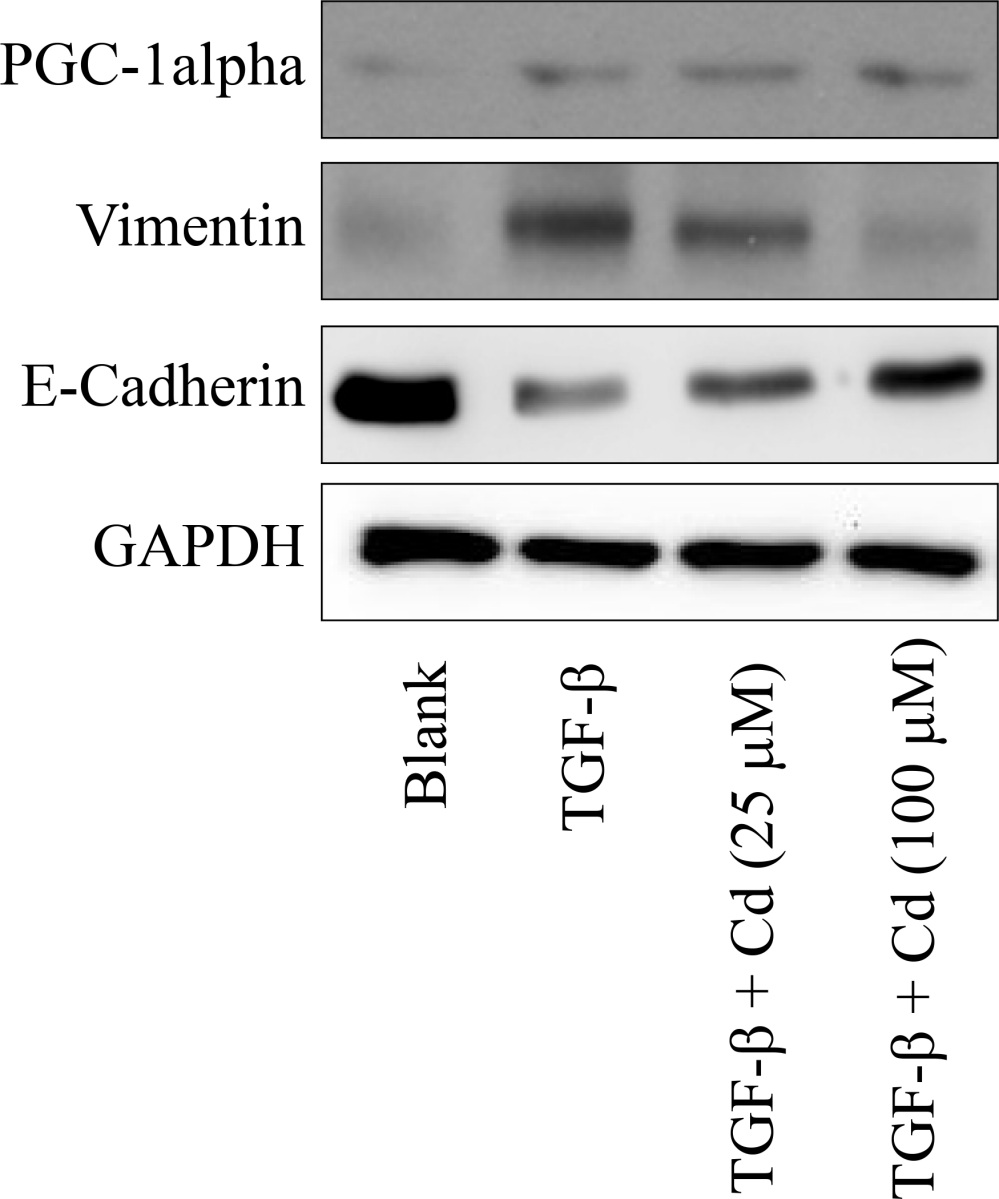
The effects of cordycepin (Cd) on PGC-1alpha and EMT markers (vimentin and E-cadherin) in TGF-beta-induced SKOV-3 cancer stem cells SKOV-3 cells were treated with TGF-beta (20 ng/mL) every 24 h for five times (5 days) with or without cordycepin (Cd). Data was repeated for 3 times.

EMT is a process that boosts invasive cells enter into the blood stream [22]. Cancer stem cells undergo EMT to migrate [23]. During EMT, the levels of E-cadherin, occludins, claudins, and desmoplakin are decreased, and the levels of vimentin, N-cadherin, fibronectin, and alpha-smooth muscle actin are incresed [24]. Recently, we have found a cotranscription factor peroxisome proliferator-activated receptor-gamma coactivator (PGC)-1alpha is associated with the metastasis of ovarian cancer [25]. In this study, we evaluated the effects of Cd on EMT and PGC-1alpha in TGF-beta-induced SKOV-3 cancer stem cells. As shown in Figure 5, TGF-beta treatment significantly increased the expression of vimentin and PGC-1alpha, and decreased E-cadherin levels in SKOV-3 cancer stem cells. However, Cd (100 μM) treatment recovered E-cadherin levels and inhibited vimentin levels in TGF-beta-induced SKOV-3 cancer cells, while there were no effects of Cd on PGC-1alpha.

### Cordycepin (Cd) attenuated chemoresistance in TGF-beta-induced cancer stem cells

**Figure 6:**
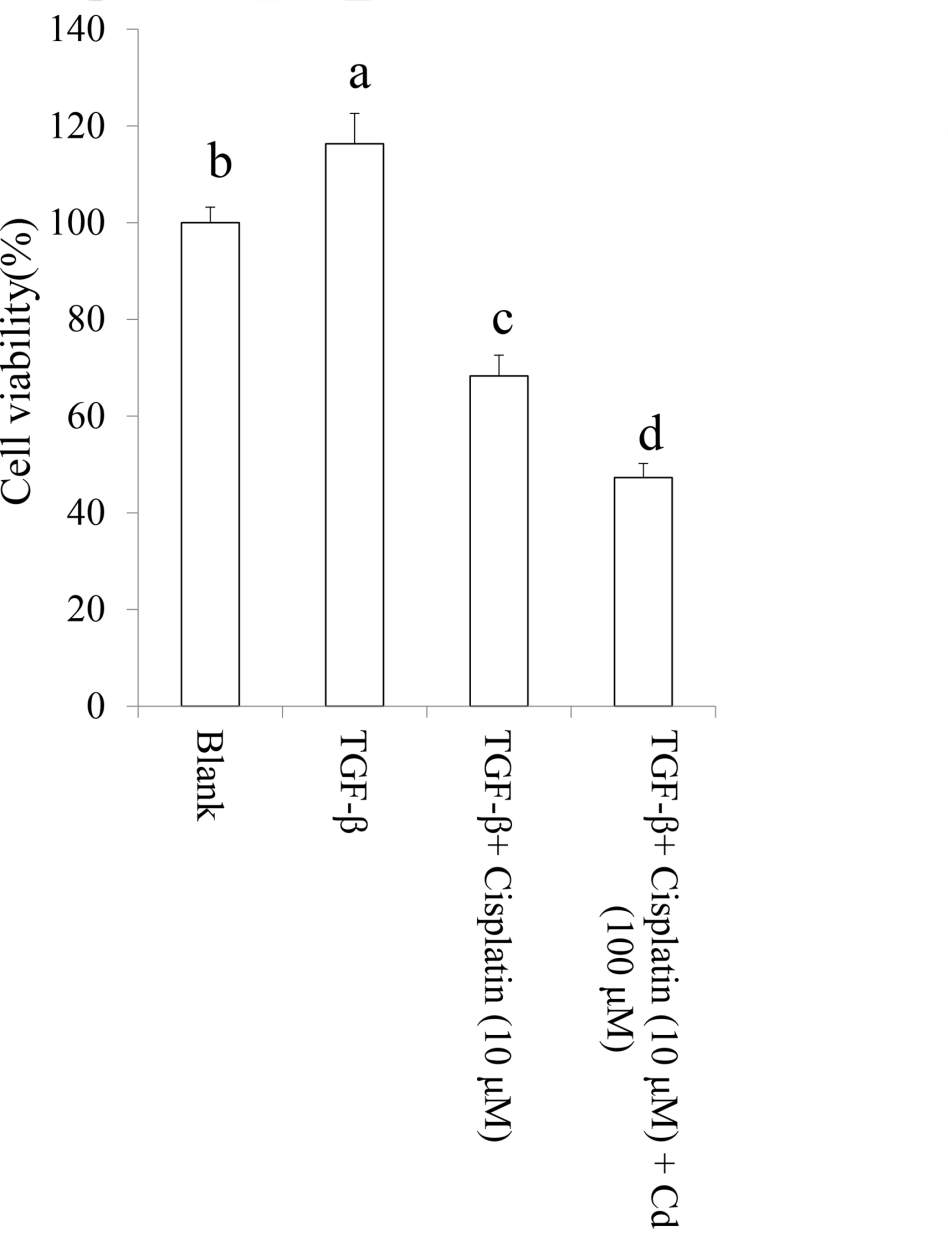
Cordycepin (Cd) attenuated chemoresistance in TGF-beta-induced cancer stem cells SKOV-3 cells were treated by TGF-beta (20 ng/mL) every 24 h for five times (5 days) with or without Cd. Subsequently, the CD44^+^/CD117^+^ cells were collected and treated with or without cisplatin for 24 h. Data was shown by mean ± SD (n = 3). **(a.b.c.d)** values with one different letter superscript are significantly different from each other (p<0.05).

The data revealed that TGF-beta-induced chemoresistance of cisplatin may contribute to cancer stemness. We investigated the effect of Cd on TGF-beta-induced chemoresistance in SKOV-3 cancer stem cells with CD44^+^ (positive) and CD117^+^ (positive) population. As shown in Figure 6, we found that Cd efficiently attenuated chemoresistance caused by TGF-beta in SKOV-3 cancer stem cells to promote the cytotoxicity of cisplatin.

## DISCUSSION

Ovarian cancer is a common gynecologic cancer with the highly mortality rate. Upon diagnosis more than 70% of ovarian cancer cases are in the advanced stages; currently nonsurgical therapies like chemotherapy and radiotherapy are the main approaches for ovarian cancer [1]. However, the development of chemoresistance against conventional chemotherapy poses a fundamental complication. TGF-beta plays important roles on cancer stemness and chemoresistance [17, 18]. Here we used TGF-beta to induce chemoresistance, which impaired the cytotoxicity of chemotherapeutic drug cisplatin in human ovarian cancer cells.

Ovarian cancer is a heterogeneous disease divided into three subtypes: epithelial carcinomas, stromal carcinomas, and germ cell tumors [2]. The invasive activity of epithelial tumor cells is based on single-cell migration, such as mesenchymal-type movement [5]. EMT is characterized by a morphological and functional shift from epithelial cells to fibroblast-like cells; this shift leads to the loosening of intercellular junctions and increase cellular mobility [26]. E-cadherin is one of the major cell adhesion molecules that form intracellular adhesion junctions in epithelial cells, the loss of E-cadherin level has been considered as the first stage of cancer cell metastasis [27]. EMT plays an essential role in cancer cell invasion and metastasis [27], and many different biomarkers have been identified to involve in EMT, such as E-cadherin, N-cadherin, fibronectin, and vimentin [27]. Previous study suggests that reduced E-cadherin levels may enhance EMT and increase the migration of cancer cells [25]. In addition, a decreased E-cadherin level is associated with poor prognosis in cervical cancer patients [16]. Human cancer cells those undergo EMT exhibit stem cell-like properties and increase metastatic potential [27].

In previous study, we demonstrate that Cd inhibits metastasis through down-regulatiing mitochondrial activity of estrogen-related receptor in human ovarian carcinoma cells [16]. However, the effects of Cd on stemness in ovarian cancer cells remain unclear. As our results, Cd attenuated TGF-beta-induced EMT and cancer stemness in SKOV-3 cancer cells. Moreover, we also found that Cd treatment suppressed chemoresistance of cisplatin caused by TGF-beta. Similarly, a study also reports that Cd disrupts leukemia stem cell activity [28].

The intrinsic resistance of CSCs, also known as tumor-initiating cells (TICs), to conventional therapy is currently regarded as a potential therapeutic target. For instance, it has recently been reported that the high rates and patterns of therapeutic failure observed in ovarian cancer are closely associated with stable accumulation of drug-resistant CSCs [29]. Because of its wide applications in cancer prevention and treatment, public interest in complementary and alternative medicine has been increased worldwide. Cd is one of the most common and crucial types of complementary and alternative medicine. Novel molecular prognostic markers, which participate in specific pathways are involved in cervical cancer tumorigenesis and tumor progression. Taken together, this study confirmed that Cd acted as a complementary agent for ovarian cancer therpy that against chemoresistance.

## MATERIALS AND METHODS

### Reagents

Crystal violet, sodium dodecyl sulfate (SDS), bovine serum albumin (BSA), insulin, Triton X-100, trypsin, cordycepin, and trypan blue were purchased from Sigma Chemical Co. (St. Louis, MO, USA). Moreover, fetal bovine serum (FBS) was purchased from Life Technologies (Auckland, New Zealand), and dimethyl sulfoxide was purchased from Wako Pure Chemical Industries (Saitama, Japan). Vimentin antibody, E-cadherin antibody, and GAPDH antibodies were purchased from Santa Cruz (Santa Cruz, CA, USA). The PGC-1alpha antibody was obtained from Novus (Littleton, CO, USA). TGF-beta was purchased from Preprotech (London, UK). CD44-FITC antibody and CD117-PE antibody were purchased from (eBioscience, CA, USA). Epidermal growth factor and fibroblast growth factor were purchased from Invitrogen (CA, USA).

### Cell culture

Human ovarian SKOV-3 carcinoma cell line was grown in Dulbecco’s modified Eagle medium (Gibco BRL, Grand Island, NY, USA) containing 2 mM L-glutamine and 1.5 g/L of sodium bicarbonate, supplemented with 10% FBS (Gibco BRL) and 2% penicillin–streptomycin. The cells were cultured in a humidified incubator at 37°C under 5% CO_2_.

### Collection of cancer stem cells

The CD44^+^CD117^+^cells were sorted from the SKOV-3 cell line by using the magnetic-activated cell sorting (MACS, Miltenyi Biotec., Bergisch Gladbach, Germany). First, SKOV-3 cells were treated by TGF-beta (20 ng/mL) every 24 h for five times (5 days), and then the CD44^+^subsets were isolated by using CD44 antibody coupled to magnetic microbeads (Miltenyi Biotec., Bergisch Gladbach, Germany) and followed by the magnetic column selection or depletion. Second, resulting cells were then depleted of CD117^−^ subsets by using mouse antihuman CD117 antibody coupled to magnetic microbeads (Miltenyi Biotec., Bergisch Gladbach, Germany). The isolated cells were placed in stem cell culture medium by resuspension in serum-free DMEM/F12 supplemented with 5 μg/mL insulin (Sigma-Aldrich, Missouri, USA), 20 ng/mL human recombinant epidermal growth factor (Invitrogen, CA, USA), 10 ng/mL basic fibroblast growth factor (Invitrogen, CA, USA) and 0.5% bovine serum albumin [30]. And the CD44^+^CD117^+^ cells were further identified by using flow cytometer (FCM, Beckman Coulter, USA). Subsequently these cells were treated with various samples (cordycepin or cisplatin) for 24 h and the cell viability was measured.

### Cell viability

The cytotoxic effect of cordycepin (Cd) on ovarian CD44^+^CD117^+^ cells isolated from SKOV-3 cells was measured using crystal violet staining assay. The cells were seeded on 24-well plates (3 × 10^4^ cells/well) and treated with various concentrations of cordycepin for 24 h. The medium was subsequently removed, and the cells were washed with phosphate-buffered saline (PBS), stained with 2 g/L of crystal violet in phosphate-buffered formaldehyde for 20 min, and washed with water. Crystal violet bound to the cells was dissolved in 20 g/L of SDS solution, and the corresponding absorbance was measured at 600 nm [31].

### Western blot

The cells were rinsed with ice-cold PBS and lysed using RIPA lysis buffer with protease and phosphatase inhibitors for 20 min on ice. The cells were then centrifuged at 12,000 ×g for 10 min at 4°C. Protein extracts (20 μg) were resolved through SDS–polyacrylamide gel electrophoresis (200 V, 45 min). The protein bands were electrotransferred to nitrocellulose membranes (80 V, 120 min). The membranes were subsequently treated with a 5% enhanced chemiluminescence (ECL) blocking agent (GE Healthcare Bio-Sciences) in saline buffer (T-TBS) containing 0.1% Tween-20, 10 mM Tris-HCl, 150 mM NaCl, 1 mM CaCl_2_, and 1 mM MgCl_2_ at a pH of 7.4 for 1 h and then incubated with a primary antibody overnight at 4°C. Subsequently, the membranes were washed three times in T-TBS, and the bound antibodies were detected using appropriate horseradish peroxidase-conjugated secondary antibodies, followed by analysis in an ECL plus Western blotting detection system (GE Healthcare Bio-Sciences).

### Measurements for matrix metalloproteinases (MMP)-2, -7, and-9 levels

Total RNA was obtained using the Trizol reagent (Gibco BRL Life Technologies, Inc., Gaithersburg, MD, USA) according to the manufacturer’s instructions. Primers were synthesized by MD-Bio, Inc. (Taipei, Taiwan). The gene expression level was determined through relative quantitative real-time PCR (CFX Cycler System, Bio-Rad Laboratories, Inc., Hercules, CA, USA.

### Statistical analysis

The analysis of variance was used to evaluate the significance of the differences between factors and expression. The means were compared using Student’s *t* test to identify significant differences among groups. The least significant difference was set at *p* < 0.05.

## CONCLUSIONS

In this study, we used TGF-beta to induce chemoresistance of chemotherapeutic drug cisplatin in SKOV-3 ovarian cancer cells. Cd treatment inhibited the cell viability, decreased the percentage of cancer stem cells, and reduced MMPs levels in TGF-beta-induced SKOV-3 cells. Treatment of Cd also regulated EMT markers in SKOV-3 cells that affecting by TGF-beta induction. Cd efficiently attenuated chemoresistance caused by TGF-beta in SKOV-3 cancer stem cells to promote the cytotoxicity of cisplatin. Taken together, Cd attenuated cancer cell stemness and chemoresistance induced by TGF-beta and the mechanism of action of Cd on ovarian cancer may contribute to regulate epithelial-mesenchymal transition (EMT) markers in ovarian cancer cells.
